# A genome-wide association study suggests that a locus within the ataxin 2 binding protein 1 gene is associated with hand osteoarthritis: the Treat-OA consortium

**DOI:** 10.1136/jmg.2009.067314

**Published:** 2009-06-24

**Authors:** G Zhai, J B J van Meurs, G Livshits, I Meulenbelt, A M Valdes, N Soranzo, D Hart, F Zhang, B S Kato, J B Richards, F M K Williams, M Inouye, M Kloppenburg, P Deloukas, E Slagboom, A Uitterlinden, T D Spector

**Affiliations:** 1 Department of Twin Research & Genetic Epidemiology, King’s College London, UK; 2 The Department of Internal Medicine, Erasmus MC, Rotterdam, the Netherlands; 3 Sackler Faculty of Medicine, Tel Aviv University, Israel; 4 Section Molecular Epidemiology, Leiden University Medical Center (LUMC), the Netherlands; 5 The Wellcome Trust Sanger Institute, Wellcome Trust Genome Campus, Hinxton, UK; 6 Department of Rheumatology and Clinical Epidemiology, Leiden University Medical Center (LUMC), the Netherlands

## Abstract

To identify the susceptibility gene in hand osteoarthritis (OA) the authors used a two-stage approach genome-wide association study using two discovery samples (the TwinsUK cohort and the Rotterdam discovery subset; a total of 1804 subjects) and four replication samples (the Chingford Study, the Chuvasha Skeletal Aging Study, the Rotterdam replication subset and the Genetics, Arthrosis, and Progression (GARP) Study; a total of 3266 people). Five single-nucleotide polymorphisms (SNPs) had a likelihood of association with hand OA in the discovery stage and one of them (rs716508), was successfully confirmed in the replication stage (meta-analysis p = 1.81×10^−5^). The C allele conferred a reduced risk of 33% to 41% using a case–control definition. The SNP is located in intron 1 of the *A2BP1* gene. This study also found that the same allele of the SNP significantly reduced bone density at both the hip and spine (p<0.01), suggesting the potential mechanism of the gene in hand OA might be via effects on subchondral bone. The authors' findings provide a potential new insight into genetic mechanisms in the development of hand OA.

Osteoarthritis (OA) is the commonest form of arthritis and a leading cause of musculoskeletal disability in middle-aged and older people.^1^ The hand is one of the most commonly affected joints in OA. Hand OA is more common in women and is significantly associated with functional impairment and reduced independence.^2^ Although it has been associated with age and environmental factors such as occupation, hand OA has a significant genetic component, with a heritability estimate of 65% estimated from one twin study.^3^ There have been several genome-wide linkage scans reporting suggestive linkage regions on several chromosomes, but only two genes (*AGC1*^4^ ^5^ ^6^ and *HFE*^7^ ^8^) have been reported to be associated with hand OA in at least two independent samples. However, inconsistent case definitions and sample sizes make the interpretation of the results inconclusive.

Genome-wide association study (GWAS) is a powerful tool for unlocking the genetic basis of complex diseases such as hand OA. The approach has been used successfully in several common diseases.^9^ Notable advantages include its comprehensiveness and the potential for finding susceptibility genes with previously unknown loci and relationship to the disease. In the current study, we carried out this GWAS for hand OA.

As a discovery sample, we used 2277 people of reputed European ancestry (1073 singletons and 602 dizygotic (DZ) twins) from the TwinsUK registry genotyped using the Hap317K chip (Illumina, San Diego, California, USA). We applied strict quality control at both individual and single-nucleotide polymorphism (SNP) levels. We excluded 51 subjects due to non-European ancestry and 3366 SNPs due to the call rate <95%, minor allele frequency <1% or Hardy–Weinberg equilibrium p<1×10^−4^ (details are provided in supplementary methods available online). After the quality control, 305 811 autosomal SNPs were available for 2226 subjects (1046 singletons and 590 DZ twins) were available. Of these, 799 women (mean age 54 years) had radiographs available for both hands. The distal interphalangeal (DIP), proximal interphalangeal (PIP), metacarpophalangeal (MCP) and first carpometacarpal (CMC) joints of the thumb were assessed for radiographic OA according to the Kellgren–Lawrence (KL) score using a standard atlas^10^ (details are provided in supplementary methods available online). We summed each joint’s KL score and used the total hand KL score as the outcome measure of hand OA. We adjusted the total hand KL score for age using regression model and used the normalised residuals as a quantitative measurement of hand OA. We then performed the GWAS analysis using the score test implemented in Merlin,^11^ which took account of relatedness (details in supplementary methods available online). None of the tested SNPs achieved sufficiently small p values to be considered significant genome-wide with a conservative Bonferroni correction for multiple testing. We therefore selected the top 100 SNPs (supplementary table 1 available online) with p⩽3.6×10^−4^ and sought confirmation in an available cohort with both genotype and hand OA data (a subset of the Rotterdam Study). The quantile–quantile (Q-Q plot) for p values (fig 1) indicated that SNPs with p⩽3.6×10^−4^ are likely to be real genetic associations because the observed p values deviate from the expected p values.

**Figure 1:**
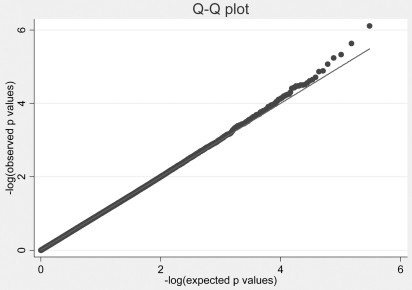
Quantile–quantile plot for the genome-wide association study in the TwinsUK cohort.

The Rotterdam Study is a prospective population cohort of Dutch men and women aged ⩾50 years. There were initially 1005 women with hand OA data available from the cohort genotyped using the Illumina Hap550K array (details are provided in the supplementary methods online). The DIP, PIP and MCP joints and the first CMC joints of both hands were assessed using the KL scoring system, and the total KL score after adjustment for age was used as in the discovery cohort analysis. Five of the SNPs we selected (rs1334995, rs2443547, rs1958654, rs938076 and rs716508) on five chromosomes were confirmed with p⩽0.05 in the Rotterdam sample (table 1).

**Table 1:** Association of the SNPs and hand OA in the discovery samples

SNP	Chr	position	Minor allele	TwinsUK cohort			Rotterdam discovery subset		
				MAF	β (SE)	p Value†	MAF	β* (SE)	p Value
rs1334995	1	70839082	A	0.18	−0.27 (0.07)	2.3×10^−4^	0.19	−0.12 (0.06)	0.05
rs2443547	5	18209429	G	0.46	0.20 (0.06)	3.6×10^−4^	0.49	−0.11 (0.46)	0.02
rs1958654	14	5329267	T	0.14	0.30 (0.08)	2.4×10^−4^	0.13	−0.13 (0.07)	0.05
rs938076	15	23784012	A	0.46	0.22 (0.05)	3.7×10^−5^	0.48	−0.01 (0.04)	0.01
rs716508	16	6276913	C	0.31	−0.24 (0.06)	6.4×10^−5^	0.33	0.10 (0.05)	0.04

β, regression coefficient; Chr, chromosome; MAF, minor allele frequency; SNP, single-nucleotide polymorphism

*Expressed as changes in total KL score per copy of the risk allele after adjustment for age

†Not Bonferroni corrected but adjusted for the relatedness in the TwinsUK cohort.

However, the effect direction of these five SNPs was not the same between the TwinsUK cohort and the Rotterdam discovery cohort for four of the five. To exclude spurious associations, we genotyped all five SNPs in another independent sample, the Chingford study, which is a well-described 20 year prospective population-based longitudinal study of osteoarthritis and osteoporosis, comprising 1003 women aged ⩾43 years at entry derived from the age/sex register of a large general practice in Chingford, North London, who are seen annually and have been described in detail previously.^12^ ^13^ The presence of hand OA was assessed in the same manner as in our TwinsUK cohort and was performed by the same assessor (DH). The analysis was performed for 637 women with both hand OA and genotype data available. Of the five SNPS, rs716508 was significantly associated with hand OA (p = 0.004) (table 2). The significance persisted even after Bonferroni correction for multiple testing in this replication sample. The other four SNPs were not significant (p = 0.20 to 0.96).

**Table 2:** Association of the SNP rs716508 and hand OA in replication samples

	rs716508		
	MAF	β (SE)	p Value
Chingford study	0.31	−0.18 (0.06)	0.004
CSAS	0.29	−0.25 (0.10)	0.009
GARP study	0.33	−0.10 (0.08)	0.20
Rotterdam study (rss)	0.34	−0.08 (0.04)	0.07
Meta-analysis	–	−0.12 (0.03)	1.81×10^−5^

β, regression coefficient; Chr, chromosome; CSAS, Chuvasha Skeletal Aging Study; GARP, Genetics, Arthrosis, and Progression; MAF, minor allele frequency; rss, replication subset; SNP, single-nucleotide polymorphism.

To further confirm this significant association, we replicated the analysis using three other samples (the Chuvasha Skeletal Aging study, the remainder of the Rotterdam cohort and the GARP Study; more details about these three cohorts in supplementary materials online). The significance was strongest in the Chuvasha study but borderline in the Rotterdam remaining sample (table 2). Although the results in the GARP study were not significant, a similar effect was obtained (table 2). The non-significant results in the GARP study might be due to the association being gender-specific, as the significant results were found in all the female cohorts (the TwinsUK, the Rotterdam discovery sample, the Chingford study and the Chuvasha study). Indeed, when we restricted the analysis in GARP only to women, the p value tended to be smaller (p = 0.10) and the effect size became larger (β = −0.14). However, the association was significant in men (p = 0.039) but not in women (p = 0.485) in the Rotterdam replication sample. An independent study is needed to clarify the sex-specific effect. Another possible explanation is simply small sample size.

We meta-analysed the summary results for the SNP rs716508 using both discovery cohorts and replication cohorts with a fixed effect model and inverse-variance weighted averages of β coefficients. The pooled effect of allele C, which is the minor allele, was associated with −0.09 grade of the total KL score after adjustment for age (95% CI −0.14 to −0.05) with a p value of 4.75×10^−5^. There was a significant between-study heterogeneity (p<0.001). This is mostly due to the Rotterdam subset used in the discovery stage, for which the effect direction of the C allele was opposite to the other five samples. The reason for this discrepancy is unclear; possible explanations include chance or random effects. When we excluded the discovery samples, the pooled effect estimate was −0.12 (95% CI −0.18 to −0.06) with p = 1.81×10^−5^ (table 2) (supplementary fig 1 available online) and there was no between-study heterogeneity (p = 0.29).

In addition, we examined the association between rs716508 and hand OA in a case–control fashion using the TwinsUK and the Chingford cohorts who had radiographs available, read by the same observer. When we categorised subjects as having hand OA if they had at least two hand joints affected, defined as KL ⩾2, the C allele was associated with a 33% reduction of risk in the development of hand OA (p = 2.0×10^−4^). The protective effect was increased to 41% when cases were defined more severely as at least three joints affected (p = 1.0×10^−5^). Similar results were obtained when the discovery sample was excluded.

The SNP rs716508 is located in the intron 1 of the ataxin 2-binding protein 1 gene (*A2BP1*) (supplementary fig 2 available online). *A2BP1* has an RNP motif^14^ that is highly conserved among the RNA-binding proteins. This protein binds to the C-terminus of ataxin-2 and may contribute to the restricted pathology of spinocerebellar ataxia type 2 (SCA2). Ataxin-2 is the gene product of the *SCA2* gene, which causes familial neurodegenerative diseases. Ataxin-2 binding protein 1 and ataxin-2 are both localised to the trans-Golgi network. Four alternatively spliced transcript variants have been found for this gene. The *A2BP1* gene has been reported to be associated with autism in a subset of patients^15^ and with smoking cessation.^16^ However, there are no reports of any association between the *A2BP1* gene and OA to date.

The *A2BP1* gene has been reported to be a novel transcriptional regulator that mediates the neuron-specific splicing pattern of the calcitonin–calcitonin gene-related peptide (CGRP) pre-mRNA.^17^ Immunohistochemical phenotypic characterisation of skeletal nerve fibres found expression of a restricted number of neuropeptides including CGRP, and osteoblasts and osteoclasts express functional receptors for CGRP,^18^ suggesting potential pathways for the association between A2BP1 and hand OA. To support this hypothesis, we examined the association between the SNP rs716508 and bone mineral density (BMD) in the TwinsUK cohort of 2094 women and found that the C allele, which is the protective allele for hand OA, was associated with decreases in BMD at the lumbar spine and femoral neck of −0.07 to −0.08 g/cm^2^ (p = 0.01 and p = 0.003, respectively). The effects became even larger after adjustment for weight.

In addition, the *A2BP1* gene is abundantly expressed in skeletal muscle, and hand-grip strength has also been reported to be associated with hand OA.^19^ It is also possible that the association between the SNP and hand OA is via muscle strength. However, the SNP was not associated with hand-grip strength (p = 0.20) or the lean mass measured by dual energy x-ray absorptiometry (p = 0.52) in the TwinsUK cohort.

In summary, we have identified a novel SNP within a gene (*A2BP1*) on chromosome 16p13.3, which is associated with hand OA in multiple independent Caucasian samples, suggesting that the findings are very unlikely to be false positives. We speculate that the potential mechanism for the association is via subchondral bone. The hypothesis is supported by the significant association between the SNP and the BMD at the hip and lumbar spine. It is known that high BMD is associated with the development of OA. The C allele of the SNP is associated with reduced BMD at both the hip and spine, and has a protective role in hand OA.

However, we did not find any association between the SNP and hip/knee OA in the Chingford cohort, the GARP study or the Rotterdam cohort in which we had knee and hip OA data available on the same subjects (data not shown). Although the low power means we cannot exclude an effect, the findings suggest a site-specific gene for hand OA, which may be true given that previous studies found that the *HFE* gene^7^ ^8^ was associated with hand OA but not hip or knee OA,^20^ and modelling studies confirm little pleiotropy between hand and large joint sites.^21^

Given that the SNP rs716508 is located in the intron of the *A2BP1* gene, further investigations are justified to clarify the role and potential mechanism of this gene in hand OA and bone.
